# Policy and practices in primary care that supported the provision and receipt of care for older persons during the COVID-19 pandemic: a qualitative case study in three Canadian provinces

**DOI:** 10.1186/s12875-023-02135-0

**Published:** 2023-09-28

**Authors:** Jacobi Elliott, Catherine Tong, Susie Gregg, Sara Mallinson, Anik Giguere, Meaghan Brierley, Justine Giosa, Maggie MacNeil, Don Juzwishin, Joanie Sims-Gould, Kenneth Rockwood, Paul Stolee

**Affiliations:** 1 Lawson Health Research Institute, London, ON Canada; 2 St. Joseph’s Health Care London, London, ON Canada; 3 School of Public Health Sciences, University of Waterloo, Waterloo, ON Canada; 4 Alberta Health Services, Calgary, AB Canada; 5 Community Health Sciences, University of Calgary, Calgary, AB Canada; 6 Department of Family Medicine and Emergency Medicine, Faculty of Medicine, Université Laval, Laval, QC Canada; 7 SE Research Centre, SE Health, Markham, ON Canada; 8 School of Nursing, McMaster University, Hamilton, ON Canada; 9 School of Health Information Science, University of Victoria, Victoria, BC Canada; 10 Department of Family Practice, University of British Columbia, Vancouver, BC Canada; 11 Division of Geriatric Medicine, Dalhousie University, Halifax, NS Canada

**Keywords:** COVID-19, Primary care, Older adults, Health policy, Virtual care

## Abstract

**Background:**

The effects of the COVID-19 pandemic on older adults were felt throughout the health care system, from intensive care units through to long-term care homes. Although much attention has been paid to hospitals and long-term care homes throughout the pandemic, less attention has been paid to the impact on primary care clinics, which had to rapidly change their approach to deliver timely and effective care to older adult patients. This study examines how primary care clinics, in three Canadian provinces, cared for their older adult patients during the pandemic, while also navigating the rapidly changing health policy landscape.

**Methods:**

A qualitative case study approach was used to gather information from nine primary care clinics, across three Canadian provinces. Interviews were conducted with primary care providers (n = 17) and older adult patients (n = 47) from October 2020 to September 2021. Analyses of the interviews were completed in the language of data collection (English or French), and then summarized in English using a coding framework. All responses that related to COVID-19 policies at any level were also examined.

**Results:**

Two main themes emerged from the data: (1) navigating the noise: understanding and responding to public health orders and policies affecting health and health care, and (2) receiving and delivering care to older persons during the pandemic: policy-driven challenges & responses. Providers discussed their experiences wading through the health policy directives, while trying to provide good quality care. Older adults found the public health information overwhelming, but appreciated the approaches adapted by primary care clinics to continue providing care, even if it looked different.

**Conclusions:**

COVID-19 policy and guideline complexities obliged primary care providers to take an important role in understanding, implementing and adapting to them, and in explaining them, especially to older adults and their care partners.

**Supplementary Information:**

The online version contains supplementary material available at 10.1186/s12875-023-02135-0.

## Introduction

The onset of the pandemic made clear that older adults are more susceptible than other sub-populations to the worst outcomes of infection [1]. As of July 2022, 92% of the 42,000 COVID-19 deaths in Canada were amongst those aged 60+ [2]. While much attention through the COVID-19 pandemic has been paid to hospitals [3–5] and the humanitarian crisis in many long-term care facilities [6–8], less attention has been paid to primary care clinics, which rapidly changed their modes of care delivery, everyday clinic practices, and their approaches to caring for older adult patients [9, 10]. Each step in this evolution of primary care was informed by local, provincial, and national policies.

### COVID-19 and primary care

The effects of COVID-19 were felt throughout the health care system, from Intensive Care Units (ICU) through to home and community care [11]. Zeber and Khanna (2021) have described its substantial impact on primary care, given the rapid transition to virtual care, decreased volumes and revenue in clinic, and the loss of preventive care and services (see also Stephenson et al., 2021) [12]. Most cases of COVID-19 were managed in the community by primary care [4]. Research conducted with primary care teams in Canada during the pandemic has highlighted the high demand for mental health services [12, 13], a sharp decline in chronic disease management and preventive health visits [12], and the transition to virtual care, or telemedicine [14, 15]. Like many high-income countries, during the pandemic, primary care services were predominantly delivered virtually [12, 16], which can include telephone visits and video calls. Specific to older adult patients, much of what has been written in the context of the pandemic has focused on the transition to, and impact of, virtual care, both in Canada [17–19] and elsewhere [20–22]. As an important health sector, primary care should continue to play a central role in current and future pandemic responses [9, 10, 23].

### COVID-19 and health policy

The term health policy has various definitions; for this work, the following definition guided this work: “*Health policy covers courses of action (and inaction) that affect the set of institutions, organizations, services and funding arrangements of the health care system*” [24]. While many papers and online tools have outlined and compared Canadian COVID-19 policies and timelines [11, 25, 26], comparatively less attention has been paid to how individuals (e.g., primary care providers or patients) experienced and navigated these policies in the provision and/or receipt of care. As is widely recognized, the development and implementation of a policy does not necessarily mean the policy was experienced or interpreted as intended [24, 27]. To understand primary care’s response to the COVID-19 pandemic and associated policies, it is essential that we hear from the providers and patients most intimately impacted (see also Matthews et al., 2021) [28]. Drawing on the phenomenological work of Workman (1992), we define a “policy experience” as the ways in which individuals receive, understand, interpret, respond to and/or enact (or do not enact) policies that relate to their personal and/or professional lives (which are often intertwined) [29].

Within this paper, we aim to examine how primary care in three Canadian provinces responded to changing health policies during the pandemic, as well as the experiences of their older adult patients during this time. Specifically, this study aimed to:

- determine how the targeted primary care clinics responded to the needs of their older adult patients during the pandemic;
- understand how primary care adapted to the ever-changing policy landscape.
- examine older persons’ experiences of receiving primary care in these clinics during the pandemic; and.
- understand the role of government COVID-19 policies and mandates on the provision and receipt of primary care for older persons.

## Methods

We conducted a qualitative case study [30], to describe how primary care providers and older adult patients experienced the changes in primary care during the first year and a half of the COVID-19 pandemic, with each case being a primary care clinic.

### Setting & context

This study was conducted with primary care providers and patients in primary care clinics across Canada; three in Ontario, one in Quebec, and four in Alberta. Clinics are located in both urban and rural communities and represent both small and large team-based primary care clinics. All clinics operated under a ‘team’ model, with more than one primary care physician and other health care professionals (e.g. nurse practitioners, registered nurses, physiotherapists, pharmacists, dietitians, etc.), and administrative staff. Eligible patient participants were older adults (aged 70+) able to provide informed consent, and able to complete semi-structured interviews in either English or French, Canada’s two official languages.

### Recruitment

We sought to interview primary care providers at the participating sites, and older adult patients who had participated in prior research conducted by the research group [31] and had provided permission to be contacted again. Researchers contacted perspective participants and informed them about the current study, provided a letter of information, and, if they were interested, obtained consent. This study has received ethics clearance and approval from the host academic institutions: University of Calgary (REB17-0617), University of Waterloo (ORE#31214) and Université Laval (#MP-13-2019-1500 and 2017-2018-12-MP). All methods were carried out in accordance with relevant guidelines and regulations. The information participants received about the study included an explanation that results would be published. Informed consent was obtained from all subjects and/or their legal guardians.

### Data collection

Data were collected using qualitative interviewing approaches by Master’s and PhD-trained researchers. Older adult participants had no relationship with the researchers conducting the interviews. Some primary care providers may have known the researchers through the previous study mentioned above, but only in the context of a research project. All interviews were conducted over the telephone or using videoconferencing software, such as Microsoft Teams. The same semi-structured interview guide was used at all sites, and sample questions are summarized in Table 1. All members of the research team contributed to the development of the interview guide, and it was modified through an iterative process, following interviews with the first two participants. Interviews were conducted from Fall 2020 through to Fall 2021. Interviews ranged from 10 to 50 min in length and were digitally recorded and transcribed by study team members. Saturation was reached in each province.

**Table 1:** Sample interview guide questions

Sample Interview Guide Questions- all sites
**HCP Interview Questions**: • Can you tell me about your experience with the various public health policies/ measures put into place to minimize the spread of COVID-19? (e.g., stay at home orders, mandatory masking, handwashing, quarantine, physical distancing, social bubbles etc.) Sample Probe: what was your main source of information? • How has COVID-19 impacted routine care for your older adult patients? • What are your most pressing concerns about your older person population? • What resources did you need/acquire/put in place to support care of older persons during the pandemic?
**Older Adult Patient Questions**: • Can you tell me about your experience with the various public health policies/ measures put into place to minimize the spread of COVID-19? (e.g., stay at home orders, mandatory masking, handwashing, quarantine, physical distancing, small social gatherings, etc.) Sample Probe: what was your main source of information? • How has the COVID-19 pandemic impacted your health **care**? Consider the lock down in the Spring – was health care impacted in March – June? Is it still impacted? • What would you say ***matters most right now*** for your health and wellness? • Looking ahead what is your biggest concern or worry related to the impact of COVID-19 on your health and care?•

### Analysis

Initial analyses of the interviews and emerging themes were completed in the language of data collection, and then summarized in English using a coding framework co-developed by the cross-provincial study team (CT, JE, SG, AG, SM, MB). Data for each participant group (i.e. primary care providers and older adults) were initially reviewed separately, and eventually common nodes were combined into the themes presented below. The coding framework was organized by the interview questions and for each participant we extracted all responses that pertained to policy at any level (clinic policies, local health authorities, provincial, federal, and others). This approach allowed researchers in each province to summarize and compare COVID-19 policy and primary care experiences. It is of note that participants rarely explicitly spoke of “policy”, and researchers used an interpretive lens to identify policy connections in these data. Researchers in each province engaged in an iterative team-based thematic analysis [32]. Transcripts were reviewed by at least two authors and agreement on themes was reached through team-based discussions. Each site prepared a high-level summary of coding nodes and sub-nodes, along with written narratives summarizing each node, and illustrative quotes to represent the most salient nodes. These provincial summary documents were then uploaded into NVivo 12 for data charting and further analysis to identify cross-cutting themes and provincial similarities and differences.

### Description of each province during the data collection period

A brief overview (see Table 2) of the policy contexts related to Primary Care and COVID-19 in each province is provided below, drawing on the timelines and tracking compiled by the Canadian Institute for Health Information [33]. This research focuses on the first three waves of the ongoing COVID-19 pandemic (March 2020 through to Fall 2021).

**Table 2:** High-level description of each province: Primary care setting and COVID-19 context

**Quebec**
• In Quebec, there are 8.6 million residents, 85% of whom are registered with a family doctor [34]. Primary health care is regulated at the central and regional levels (16 regions). GPs are mainly paid on a fee-for-service or flat-rate basis, while the other professions are paid on an hourly basis. Most GP services are provided in *Groupes de Médecine de Famille* (GMF, Family Medicine Groups). • Declared a state of emergency on March 13, 2020, and on that same day ordered the closure of all schools, post-secondary-educational institutions, and most daycares. • Order in Council 223–2020 in Quebec [35] paused non-emergent and non-urgent procedures and treatments in clinics. • March 14, 2020, the province reiterated that all residents over the age of 70 should stay at home as much as possible, except for things like medical appointments. It was also announced that the *Régie de l’assurance maladie du Québec* would cover the cost of health services offered virtually (including telephone) [36]. • For our period of data collection, Quebec experienced waves of COVID-19 that peaked in April/May 2020, January 2021, and April 2021. Throughout, the province has relied on a regional colour alert system, green (“vigilance”) being the lowest level of alert, and red (“maximum”) being the highest; each colour is associated with different restrictions and guidance related to social distancing, closures, private gatherings, visits to Long Term Care, etc.
**Ontario**
• In Ontario there are multiple primary care models, including Family Health Teams (which are often interdisciplinary), solo-practitioner and fee-for-service clinics, and Community Health Centers, that are compensated through a blended payment model (i.e., salaries based on roster size and additional fees for some services). • State of emergency was declared on March 17th, 2020, and this coincided with population-level interventions to reduce spread (e.g., closures of schools and many businesses) • Directive #2 for Health Care Providers in Ontario [37] resulted in pausing non-emergent and non-urgent procedures and treatments in clinics. Significant rise in positive case numbers during April/May 2020, January 2021, and April 2021. • ‘Digital First’ approach in Ontario [38], formal policy announcements urging physicians to use virtual care whenever possible, and the introduction of billing codes to compensate for these visits (including telephone) [33]. • Local response by the 34 Ontario public health units differed depending on the case numbers and hospitalizations. For example, Ontario used a colour coded system to communicate the level of risk in each public health unit; green (“prevent”) being the lowest risk and with the lowest restrictions on activities, and grey (“lockdown”) reflecting the highest risk and indicating to return to the strict measures put in place during the first wave to reduce transmission. • For our period of data collection, Ontario experienced waves of COVID-19 that peaked in April 2020, January 2021, April 2021, and September 2021.
**Alberta**
• In AB, primary care resides under Alberta Health (provincial Ministry of Health). Primary care clinics are owned and operated by family doctors, and most are members of a Primary Care Network (PCN), which essentially offers a multi-clinic collaboration to provide interdisciplinary programs and services based on agreed local priorities for the geographical area served. PCNs are a joint-venture agreement between physicians and Alberta Health Services (province’s single health authority) but governance and funding responsibility lie with the Ministry of Health. PCNs have been an essential component of transforming primary care in Alberta over the last two decades [39]. • State of emergency was declared on March 17th, 2020, and this was coupled with the closures of recreational facilities, and limitations on non-essential services; one day prior, on March 16th, all educational facilities were closed. Additional closures and restrictions for non-essential services were put in place two weeks later. • March 18th, 2020, family doctors were asked to switch to primarily virtual care to reduce spread, and to bill using temporary virtual care codes (which were made permanent billing codes in June 2020) [40]. Alberta Health Services announcements paused non-urgent and non-emergent primary care in clinics. • AB has experienced waves of COVID-19 that peaked in April 2020, December 2020, May 2021, and September 2021.

## Results

Overall, across the three provinces, seventeen interviews were conducted with a variety of primary care providers and 47 interviews were completed with older adult patients. Two overarching themes were identified: (1) navigating the noise: understanding and responding to public health orders and policies impacting health and health care, and (2) receiving and delivering care to older persons during the pandemic: policy-driven challenges & responses. For each theme we outline the challenges that participants identified, and share the strategies and solutions implemented to address these challenges. Theme one highlights clinic operations and policy implications on care delivery, and as a result, heavily relies on data obtained through primary care provider interviews. Older adults were asked about their experiences with provincial guidelines and policies, but the participants focused more on personal and care experiences. Theme two highlights views from both primary care providers and older adults as it relates to delivery and receipt of primary care during the pandemic.

### Theme 1: Navigating the noise: understanding and responding to public health orders and policies impacting health and health care

With national, provincial and local policies to interpret and navigate, participants discussed the challenges that they encountered in following COVID-19 policies related to primary care, and also shared the strategies that supported their efforts to interpret, follow and apply policy.

#### Volume of policies impacting health and health care

Both providers and patients emphasized that communications about pandemic related policies were constant and inconsistent (and/or could be interpreted in an inconsistent manner), and that the rapidly evolving nature of pandemic-related policies made it difficult to stay abreast of changes. As a provider in Alberta noted, *“everything changes every day”* (HCP3), and a clinician in Ontario echoed “Y*eah, it was changing daily…So, just trying to be diligent with keeping things up to date…yeah.*” (05_ON_HCP). Another provider in Ontario described the initial policy context as “shifting sand”:

*As you can well imagine, it was like walking on shifting sand [laughter] it’s there for the longest time, which was you know, it was wearing for everybody.* (**ON_HCP04)**

#### Grey areas & ambiguities

Many clinicians also described how policies can be open to interpretation at the clinic and/or provider level. For example,

*When you make a broad-brush comment or instruction, the devil is in the details. And it’s very hard to interpret. I think- you know, I heard it often from my staff too- is people want guidance, that’s black and white, and concrete; and it’s just not possible! So, every advice is open to interpretation. I’m sitting here in the clinic right now. And for example, one of our goals would be to minimize cross traffic between patients and staff, people come in one door and out another, but we can’t do that because we have four entrances that face the carpark. And if we were to have people walking through the building to come in and out of each one of those doors in a flow kind of way, we’d actually increase the amount of cross traffic.* (03_**ON_HCP**)

This quote captures a sentiment expressed across all three provinces and both primary care providers and older adults: that the policy directives and mandates were open to interpretation and/or could become sources of ambiguity rather than clarity, depending on how they were communicated. When ambiguous policies are addressing important issues of protection and disease prevention, the grey areas can become a source of stress. For example, a clinician explained that the use of masks was not always obvious:

*For sure, over time, things became clearer, but there were a few weeks where it wasn’t so obvious: “So, do we wear masks or don’t we? Do we change or do we not change? Is this okay or not?” I mean, we eventually worked things out, but there was always this kind of feeling of not being sure we were doing the right thing, what should we be doing, you know? (***QC_HCP08***).*

In Quebec, there was a general ban on visitors accompanying patients to appointments, and thus, patients were more likely to forget or misunderstand the information received. For some patients, caregivers were able to accompany older adult patients, however clinicians noted that the concept of caregiver was poorly defined and therefore they did not know who was authorized to have a caregiver support person at their in-person appointments, given the general ban on visitors. Several health professionals said they were flexible and allowed patients to be accompanied. A healthcare professional reported:

*Well, sometimes the challenge was… because patients had been told to go to their appointments on their own, except if they needed a caregiver with them, and sometimes that distinction isn’t easy to make, so since the epidemiology is favourable, we’re a little more lenient when it comes to caregivers, because sometimes when our older persons come on their own, they may not have all the background info and can have trouble following up.* (**QC_HCP06**)

#### Policies differing by regional zone

In Ontario, providers and patients described the additional policy challenges posed by the colour-coded zones and regions implemented in the Fall of 2020. Although there were also efforts to implement regionalized pandemic responses in Quebec and Alberta, this did not appear to be a source of stress or confusion for participants there, as it was not raised in their interviews. In Ontario, we heard examples such as: sometimes where a provider lived and where they provided care were in different colour-zones of pandemic response, doubling their pandemic-related guidance and increasing confusion about which set of policies to follow.

In addition, local public health units were charged with implementing policies, and in some cases clinicians and patients straddle two regions, and/or were trying to glean guidance from different sources. An executive director in Ontario provided this example about gloving policies:

*There was one about … when you should be gloving and when you shouldn’t be gloving. And then so Public Health would send something out. And what I learned later was [that they] weren’t consulting…the two public health units were saying different things.* (**ON_HCP02**)

Providers, however, also recognized that regional differences were both appropriate and to be expected. An executive director noted:

*And that’s to be expected, right? Because public health units are supposed to be responding to what’s happening in the communities they serve….And at the outset, [one local public health unit] had way more cases, so [that unit] was putting on stricter controls.***(ON_HCP02)**.

In navigating the noisy and complex policy environment outlined above, clinics also implemented or benefited from strategies put in place to support the streamlining and clarification of information.

#### Responses and strategies to support communication and clarity

While older adults received most of their policy information from the mainstream media, and on occasion social media, providers more heavily relied on their local public health units and health care governing bodies for policy communications and guidance. Professional associations, such as the Quebec Federation of General Practitioners, were also key and trusted sources of information. In Alberta, providers appreciated the coordinated approach implemented by Alberta Health Services and the PCNs. We interviewed clinics in two PCNs; one PCN was particularly effective and proactive in disseminating relevant policy updates and information from Alberta Health Services and Alberta Health, highlighting what was important for primary care. The support nurses and executive directors then communicated this to the clinics:

*AHS [Alberta Health Services]… I don’t know if they still are but was putting out a weekly summary of new directives and directions. Then our executive director would forward those out to us and highlight anything that was pertinent to us. So, we always had current information on changes, what was happening, direction from our leadership.* (**AB_HCP02**)

This consolidation of trusted and relevant policy information was highly valued, as described by an administrator in Ontario:

*So we would use Public Health Agency of Canada, Ontario Public Health, and then the community health centers have a sort of overarching organization called the Alliance for Healthier Communities. And the Alliance did a wonderful job …reviewing all of the information that was coming in from the two government agencies, and sort of summarizing them and making relevant documents available to us. So we didn’t have to go hunting through things to get the relevant information. So that was helpful.***(ON_HCP01)**.

Clinics employed a number of strategies to help manage both the volume and confusion around incoming policies. Strategies included: appointing one staff member to track incoming policy communications, engaging in regular (up to three times/week) clinic huddles to review incoming policies and risk mitigation strategies, and offering extra educational sessions to review new policies. These were powerful; one provider in Alberta said that extra education sessions provided to address questions were one of the most useful parts of their local COVID response. The questions were ‘community generated’ and she felt she learned more through that than anything else.

*They actually did an afternoon information session, answering all of our questions so we could leave there feeling informed and also so we could educate our patients about some of the misconceptions or interpretations or just where they were getting their information … It was just more about empowering patients…that is where we got our biggest amount of information and I feel like I learned, more than I would have learned on any website … It was simple.* (**AB_HCP01)**

The abundance of information and rapidly changing policy directives not only impacted the primary care clinic operations, but also influenced how providers were delivering care during the pandemic.

### Theme 2: Receiving and delivering care to older persons during the pandemic: Policy-driven challenges & responses

Within a complex and evolving policy context that dramatically impacted primary care, providers and older adults discussed how and why care for older patients was limited, and how clinicians and clinics responded.

#### Delayed and delaying care

We heard from both older adults and providers that, particularly at the beginning of the pandemic, older adults were avoiding and not accessing the health care that they required. An administrator in Ontario explained:

*Probably a majority of our visits, particularly with seniors, would have been by telephone. We did continue to see patients with urgent issues. And I know, many seniors were very reluctant to come out or to come into clinic…. We did have some concerns that seniors were not following up on things they should have followed up on. Then we had a few calls where we, you know under normal circumstances the best course of action would be to go to emerg…We had a few situations where we really had to work with family members to get people to emerg because they were just afraid to go there.* (**ON_HCP01**)

Across all sites, providers noted that they were concerned about not being able to meet the needs of their older patients who were most vulnerable; as underscored by a provider in Alberta, “*there’s a population [of seniors] that we are missing and it’s just to figure out how to get to that group.*” (**AB_HCP01**). Connecting with older persons was a challenge for clinics, as one provider explained, the clinic sometimes didn’t hear from patients aged seventy-five and older, *“they went underground, we didn’t hear from them*” **(ON_HCP02**).

We also heard from participants who delayed care as they were avoiding all interactions outside of the home. For example, an older adult participant in Alberta shared:

*… my doctor got really upset with me, and indicated how important it was to try to maintain that schedule of testing… but I was I was, actually, I was afraid to even try to access or I didn’t even know that I could because with, with the medical resources being taxed to the limit, and, and not wanting to be exposed to, you know, to the public or others, it was a lot of unknowns*. **(AB_Pt03)**

Concerningly, participants also described minimizing their needs, feeling obliged to endure pain or conditions because of the pandemic, and avoiding their physicians because they either did not want to burden them, or felt that accessing care was simply too complicated. For example:

Interviewer: *What is most important to you right now in terms of healthcare?*

Patient: *Healthcare? I don’t need healthcare. I’d just like to have a medical opinion about my arthritis that’s been acting up. But I know that’s what it is, you see. It’s not a big deal; it’s just painful. I figure if I had a medical opinion, if I had medication to relieve the pain, maybe that would help me. But I’m not going to insist on seeing someone for that. I don’t think… I don’t think it’s serious enough for that. (***QC_Pt05***)*

Outside of primary care, participants noted many delays in routine screening tests, cancelled appointments, and the postponement of surgery.

*I had a cataract surgery scheduled for October, which was cancelled and now it’s supposed to be January, but who knows? … I had a CT scan done in February. And I was supposed to see him a couple months after that, and I mean it got held up for a couple more months later*. (**AB_Pt15)**

And,

*We had really good service, but my problem is that I need injections in my spine and it’s harder to get an appointment… (…) it’s the anesthetists who give the injections. There’s a lot of demand, and when COVID really started in May at the hospital, they had to stop taking appointments (…). It was frustrating because I’d been waiting for my appointment for nearly two years, and then it got cancelled. I called, I’ve called them twice, and they don’t know when I’ll get to see him.***(QC_Pt42)**.

### Responses and strategies to support care for older persons

Recognizing that older patients were not receiving care as they normally would, providers shared a number of strategies that they employed to support these patients. Strategies included: wellness checks and making time for older patients; making the clinic a safe place; and a pivot to virtual care.

#### Seeking out and caring for older patients in the community

To address some of the unmet care needs outlined above, particularly the feeling that the patients were “*going underground*”, primary care practices in Ontario responded by engaging in regular “wellness checks”, and providers in all three provinces discussed caring for older adults in their homes and the community.

*Wellness checks and making more time for older patients.* In Ontario, participating clinics used their electronic medical records to identify patients aged 70 and older, and engaged in intentional outreach, principally over the phone. Rather than following-up on routine medical issues, the wellness checks were designed to elicit how older persons were doing and assess any unmet needs. An administrator described their wellness checks:

*We had the nurses contact all patients over the age of 70. So probably a couple of weeks into the lockdown, we were doing that reaching out to them just to see how they were doing, if they had questions, if they knew that they could still contact us…I think it was really encouraging for both seniors and the staff. The staff felt that they were being useful, and they were really appreciative of the acknowledgement they got from the seniors. And certainly, the seniors were just delighted to have someone to talk to, sometimes.”* (**ON_HCP01**).

Similarly, providers in Alberta and Quebec described taking more time with their oldest patients; for example, “*it has taken up more of my time being supportive in a social way than normally my role would require me.*” (**AB_HCP02**).

*Primary care in the home.* Across all three provinces, we heard stories of physicians and clinics going out of their way to provide care for their older patients, as demonstrated above with the examples of wellness checks and telephone-based virtual care. From the Quebec clinics, we also heard about an increase in home visits, which was requested by the province and coordinated regionally. These directives to expand home-based primary care for older persons came from the DRMG (Département régional de médecine générale) and the DSP (Direction de Santé Publique). While providers in all three provinces made mention of some home care visits, in Quebec, the delivery of primary care services in the home was a more commonly discussed change in care provision for older persons. For example, all blood tests for people over the age of 75 were taken at home and family physicians increased their number of home visits. A clinician recounted:

*It depended also on the level of vulnerability. For a number of my patients, this meant appointments ended up being in-home visits because it was just easier that way (…) If I was able to do some things over the phone then I did them over the phone, and if I needed to perform a technical act or if a physical exam was required, then sometimes I’d go and see them to do that at the end of the day or the end of the morning.* (**QC_HCP02**)

Some healthcare professionals believed that this increase in home care improved access to care for older adults and recommended that this increase be maintained even after the pandemic.

There were, however, complexities and challenges associated with this shift to care in the home, including feelings that home visits were more complex and time consuming, and that communication is trickier when providers are not in clinic.

*Making the clinic a safe space to receive and provide care.* Providers emphasized that they were able to continue providing primary care services to older persons. As a provider in Alberta described, “*I have been seeing patients through COVID, care has just looked a little bit different.*” (**AB_HCP01**). Providers described changes to clinic flow, staggered appointment times, the use of personal protective equipment (PPE; which differed at different points of the pandemic and by jurisdiction), etc. Older adults also described new procedures to accessing care at their primary care clinics, for example, calling ahead of time, completing COVID screening in the parking lot or outside the clinic, etc. One provider described this shift in care provision, and how it gave patients a sense of comfort:

*We made sure that our geriatric nurses were still seeing people face-to-face. So for instance, our INR program (anticoagulant management), we kept seeing people, but we transitioned to parking lot visits. So, our nurse would put on PPE and she would schedule people, and she would go and do the finger prick INR testing while they were in the car. And, and so people really liked that because they didn’t have to be coming into buildings, where they had to touch stuff.***(ON_HCP02)**.

While some patients found these new arrangements difficult, others appreciated having the protocols in place. This diversity of perspectives is illustrated by these two quotes from patients in Alberta:

*There’s a protocol, if you’ve got to go to the clinic, … when I went to see Dr. [name] for the first time, … I would phone them as soon as I got to the, to the [clinic name], let them know I was in the parking lot. And, and I was to stay in my vehicle. And they would contact me as soon as he had finished seeing the previous patient. And, and then that way I would go up, and I would go directly into the exam room. … I must say, is, is pretty well in place, they give you the mask, they use sanitizer.* (**AB_Pt03)**

*It is worse than getting into Fort Knox. You come up, they ask you 40 questions, all of what you say no, you come back the next day and it is the same 40 questions… then you get to go sit in a room after they call you from your car to go sit in a room.* (**AB_Pt14)**

Although the policy landscape was complex and ever-changing, providers described how these policies and profound changes to providing care felt like a source of protection: “*I think the PCN did an amazing job of being flexible and yet still protecting not only the patients, but also their employees.*” (**AB_HCP03**).

#### Pivoting to virtual care

Patients and providers both described a notable increase in the use of virtual care, or telemedicine, most of which was done over the telephone. While the challenges of using technology to do virtual care was a topic of interest for health and primary care providers, it was less so for our sample of older persons. While some participants voiced their concerns, most were generally satisfied with the quality of care they received. An example of this apprehension was expressed by a patient in Ontario:

*I’m not excited about that [telephone visits], you know, you spend so many years then having a doctor see you and assess you, things like that, and I’m just a little concerned, you know?* (**ON_Pt02**)

In our participating clinics, “virtually” often meant over the telephone, not necessarily through online methods. As an administrator in Ontario noted,

*Seniors were comfortable with telephone. And we did have the capacity to do a virtual call, but most people it was just another level of technology that that they weren’t as comfortable with.***(ON_HCP01)**.

This emphasis on telephone appointments was due to several factors: patient preference, internet connectivity challenges in rural areas, and the recognition that some of their oldest patients were not equipped or able to use some telemedicine applications. Although some participants were not keen on virtual care, they also noted that clinics were willing and able to accommodate in-person appointments when needed:

*Both my wife and I both had some online or virtual appointments…if we really needed or insisted on a face to face then they would do that.* (**AB_Pt07)**

*There’s no problem to see my family doctor. She came to see me when everything was locked down tight. She came to see me at home. After that, she wasn’t allowed to anymore, so she would do her appointments over the phone. I thought that was perfect. And the times I had to go to the hospital for tests, I thought it went fast and everything worked really, really well. (***QC_Pt30***)*

Given the ever-changing COVID-19 environment, primary care clinics and providers did their best to manage policy complexities and respond appropriately to the needs of their older adult patients.

## Discussion

Our participants, older patients and primary care providers, described a policy context that was constantly changing, and at times, difficult to navigate. Some of the policies and communications from politicians, ministers and public health officers (e.g., pausing in-person care, the shift to virtual care, older persons urged to shelter in place), while necessary to prevent the spread of infection, made it more difficult to provide primary care to older persons. This was similarly expressed in a Belgian study of primary care experiences during the pandemic, which queried whether “the cure [will] be worse than the disease” [41]. In this challenging context, the primary care participants in various study clinics shared a number of policy responses that allowed them to continue providing care as best they could to older and frail patients. In this, we see that policy can be both a source of guidance and information, and simultaneously can require additional sets of actions, responses and more policies [23].

### Supportive primary care approaches to caring for older adults

In planning for future outbreaks, and generally considering the provision of primary care to older and frail patients, clinics and providers in all three provinces shared details on the creation and implementation of local-/clinic-level policies that supported the health care of older persons. Study sites in all three provinces, but most explicitly in Ontario discussed the development of intentional and scheduled “wellness” checks for older and/or frail patients. In Alberta the PCNs and clinics played a crucial role in sorting, consolidating, and prioritizing appropriate policy directives and changes in policies, including information sessions that responded to community-generated questions (see also [23, 38]). In Quebec, there was a clear, local-level shift towards more primary care provided in the home, including in-home bloodwork for older adults. This approach may have helped address the “competing crises” [42] of both COVID-19 and acute social isolation amongst older adults, particularly when Quebecers over the age of seventy were advised to shelter in place at the outset of the pandemic [34]. These are all promising policy responses that addressed the many challenges in providing care to older persons during a pandemic [18]. These practices also echo what was also done by “high performing primary care practices” in the United States, including proactive outreach to vulnerable patients and home-based care and monitoring for the most vulnerable [43]. Proactive wellness checks, reported by Albert and colleagues (2021), are especially important, as we heard from our participants that some older patients were avoiding care and “going underground”; similar concerns about older patients were echoed in Hogan et al.’s commentary on the importance of geriatric care during the pandemic [18].

### Virtual care

As has been described by others [14, 17–19, 21, 22], our findings also highlighted the impact of virtual care for older primary care patients. Virtual care does not always mean via the computer. Many of our sites preferred the telephone, even if they were equipped with the tools to offer virtual care using videoconferencing software. As supported by others e.g., [44] virtual care may have potential to be used as a supplement to in-person visits, and should not replace face-to-face visits. Glover and colleagues (2022) have developed a tool, in alignment with Ontario’s Virtual First policy, to help providers determine if virtual care is appropriate for older adult patients [45], and this information is also echoed in Ontario’s Virtual Care Services document which highlights services and fee codes for physicians [46]. Primary care providers and clinics may need to refine their protocols for best supporting older adults, recognising the issues of accessibility and digital connectivity in rural areas for some of their patients.

### Communication within, to and from primary care

The noisy and complex policy landscape described by our participants is best illustrated by the 639 provincial policies or public health orders introduced in Quebec, 609 in Ontario, and 374 in Alberta that we counted during the time period upon which our participants reflected on (i.e., from the start of the pandemic to the second round of interviews) (*COVID-19 Intervention Timeline in Canada* [25]. These numbers do not include additional policies that may have been introduced by regulatory bodies, regional- and local-level public health units, and primary care clinics themselves. Our participants highlighted the vital importance of having instant communications from trusted sources during times of “new and emerging diseases”, a finding also highlighted by others [47–52]. Older Canadians viewed the complexity of the communication environment challenging. Clearer communications from health policy sources could help older Canadians and health and primary care providers. Learning and practicing how and when to communicate policy to the public in circumstances of uncertainty is required, and further research in this area may be needed [53].

### Limitations

We recognize that our sample was limited to primary care providers and staff attached to the participating clinics and primary care teams, and older adult patients who were attached to these clinics, previously consented to participating in research, and who spoke either English or French. Before the pandemic, an estimated 550,000 Canadians over the age of 65 did not have a primary care provider [54], and the perspectives of those patients are not reflected in this paper. In addition, we note that the voices of older adults do not come through as strongly as we would have wished or expected at the outset of data collection. Rather than discussing primary care or health policy, the most salient themes from our interviews with older persons were related to isolation, fear, and loneliness, all of which will be the focus of a forthcoming companion manuscript. Additionally, we need to acknowledge that primary care clinics were required to respond quickly to the ever-changing policy environment, as such, we may not have captured all of the COVID-19 policy implications in this study.

## Conclusion

This paper explored how primary care clinics in Alberta, Ontario, and Quebec, cared for their older adult patients (70 years of age and older) in an ever-changing health policy landscape. This paper also highlighted the experiences of older adults, clinicians, and primary care clinics navigating constant and confusing flows of information during the COVID-19 pandemic from Fall 2020 until Fall 2021, and the rapid shifts that took place to provide alternate approaches to care. Future pandemic planning should include considerations for improved dissemination of information during uncertain times, including easy-to-understand guidelines for primary care providers delivering care, and for patients receiving care.

### Electronic supplementary material

Below is the link to the electronic supplementary material.

Supplementary Material 1

## Data Availability

The data analysed during the current study are not publicly available, due to the confidential nature of participant transcript data but are available from the corresponding author on reasonable request.

## Abbreviation

ICU: Intensive Care Unit
