# Supplementary material for: Policy and practices in primary care that supported the provision and receipt of care for older persons during the COVID-19 pandemic: a qualitative case study in three Canadian provinces
Source: BMC Prim Care. 2023 Sep 28;24:199. doi: 10.1186/s12875-023-02135-0 (PMC10536733; doi:10.1186/s12875-023-02135-0)
Supplement: Supplementary file 1 — Supplementary Material 1 [file 12875_2023_2135_MOESM1_ESM.docx]

**Supplementary Materials**

**Appendix 1. Overview of coding and the identification of cross-cutting, high-level themes.**

| **Initial identification of themes for each case**  (independently coded by provincial teams) | | | **Consolidating high-level themes across the provincial cases**  (charted, consolidated in NVivo 12) | **Cross-cutting themes with policy connections** |
| --- | --- | --- | --- | --- |
| **ALBERTA**  **Nodes & sub nodes** | **ONTARIO**  **Nodes & sub nodes** | **QUEBEC**  **Nodes & sub nodes** |  |  |
|  | HCPs experiences of how policy impacted primary care   - Pandemic preparedness, PPE & covid testing - Changes within clinic (flow, timing, parking lots, costs, budgets) - Differences across regions/zones | HCPs experiences of how policy impacted primary care   - PPE - Health human resources, redeployment to other sectors & less patient time - Differences across regions/zones | **1. Navigating and implementing clinic-, regional-, and provincial-level policies**  Subtheme i: grey zones and ambiguities  Subtheme ii: responses and strategies to support communication  Subtheme iii: policies differing by regional zones | **Theme 1:**  ***Navigating the noise: Understanding and responding to public health orders and policies impacting health and health care*** |
| Policy connections, HCPs   - Policy to support coordination and action - Policy as protection   Policy communication | Policy connections, HCPs   - Achieving consensus and seeking/finding guidance   Communication |  |  |  |
| Policy connections, Older Adults   - Navigating new policies (sources, trust, noise, action)   Communication | Policy connections, Older Adults   - Sources of policy info & changes - Communication | Policy connections, Older Adults   - Sources for policy info (felt informed, but saturated, seeking trusted sources (e.g., family, HCPs) | **Note:** not a robust or rich theme, compared to others. Patient experiences of COVID-19 policies were more closely tied to themes of loneliness, isolation, fear, and less closely related to the receipt of health care. See note below about companion manuscript that addresses these themes. |  |
| Older adults’ experiences of being cared for   - Access to care & logistics - Move to virtual care - Delaying health care | Changes to care, older adults   - Shift to virtual care - Some cancelled/delayed care, but not many concerns about access or delays - Changes at clinic | Changes in healthcare, older adults   - Delayed and avoiding care, some enduring pain - Shift to telecare as main mode - Difficulties accessing care - New health needs | **2. Older adults’ experiences of receiving primary care**   - Delays in seeking and/or receiving care, in some cases - Changes in access to care and logistics, both at their primary care clinic and elsewhere in the health care system - A notable shift to virtual care/telecare, which was largely telephone-based | **Theme 2*: Receiving and delivering care to older patients during the pandemic: Policy-driven challenges & responses*** |
| HCPs experiences of caring for older adults in pandemic   - Care continued, largely satisfactorily - Virtual care - Risk for older persons | Changes to caring for older adults   - Decline of older persons, reluctance to seek care (but changed over time) - Some home care - Virtual care - Check-ins for older persons - Retirement homes problematic | Changes to caring for older adults   - Telecare main mode - Shift to more home care for older persons - Concerns about patient’s physical health and noticed declines - Concerns about older person autonomy - MDs assigned to LTC homes | **3. Providers experiences of caring for older adults**   - Emphasis on continued care, largely through virtual/telecare - Concerns about risk and declines in health amongst older persons - Changes to delivery, including check-ins for older persons in ON, increased home visits in QC |  |
| **Note: Across all cases, there was also ample discussion on how the pandemic impacted the wellbeing of both older adults and HCPs, and these data will be used in a forthcoming companion manuscript. Nodes for that coding included things like ‘changes to routines’, ‘isolation and loneliness’, ‘stress’, ‘pandemic fatigue’, ‘mental health’, ‘fear’, ‘adjusting’, and ‘resilience’.** | | | |  |
